# Comparison of Epstein–Barr virus and Kaposi’s sarcoma-associated herpesvirus viral load in peripheral blood mononuclear cells and oral fluids of HIV-negative individuals aged 3–89 years from Uganda

**DOI:** 10.1186/s13027-023-00516-9

**Published:** 2023-06-14

**Authors:** Angela Nalwoga, Vickie Marshall, Wendell Miley, Nazzarena Labo, Denise Whitby, Robert Newton, Rosemary Rochford

**Affiliations:** 1 Department of Immunology and Microbiology, University of Colorado, Anschutz Medical Campus, Aurora, CO USA; 2 MRC/UVRI and LSHTM Uganda Research Unit, Entebbe, Uganda; 3 Viral Oncology Section, AIDS and Cancer Virus Program, Leidos Biomedical Research, Inc., Frederick National Laboratory for Cancer Research, Frederick, MD USA; 4 University of York, York, UK

**Keywords:** Epstein–Barr virus, Kaposi’s sarcoma-associated herpesvirus, Viral load, HIV uninfected individuals, Across the age span, Uganda

## Abstract

We previously found that age, sex and malaria were associated with KSHV in individuals from Uganda. In this study, we have evaluated these same factors in relation to EBV in the same specimens. Overall, 74% (oral fluids) and 46% (PBMCs) had detectable EBV. This was significantly higher than observed for KSHV (24% oral fluids and 11% PBMCs). Individuals with EBV in PBMCs were more likely to have KSHV in PBMCs (*P* = 0.011). The peak age for detection of EBV in oral fluids was 3–5 years while that of KSHV was 6–12 years. In PBMCs, there was a bimodal peak age for detection of EBV (at 3–5 years and 66 + years) while for KSHV there was a single peak at 3–5 years. Individuals with malaria had higher levels of EBV in PBMCs compared to malaria-negative individuals (*P* = 0.002). In summary, our results show that younger age and malaria are associated with higher levels of EBV and KSHV in PBMCs suggesting malaria impacts immunity to both gamma-herpesviruses.

**Supplementary Information:**

The online version contains supplementary material available at 10.1186/s13027-023-00516-9.

## Introduction

Among the known human herpesviruses, the gamma-herpesviruses Epstein–Barr virus (EBV) and Kaposi’s sarcoma-associated herpesvirus (KSHV) are carcinogenic [1]. EBV is causally associated with Burkitt’s lymphoma, certain Hodgkin’s and non-Hodgkin’s lymphomas, nasopharyngeal and gastric carcinomas, while KSHV is the cause of Kaposi’s sarcoma, multicentric Castleman’s disease (MCD) and primary effusion lymphoma (PEL) [2, 3]. Of these malignancies, PEL cells are infected with both EBV (~ 80%) and KSHV (100%) [4]. In sub-Saharan Africa, both EBV and KSHV infections are very common [5]. Infection with both viruses occurs in childhood, with EBV infection occurring earlier than KSHV [6, 7]. Both EBV and KSHV have been shown to infect and establish latency in several B cell subsets [8, 9]. However, the interaction between the two viruses has not been extensively studied ex vivo.

Both EBV and KSHV viral load are accurately quantified using real time polymerase chain reaction (PCR) [10–12]. Detection of both viruses in oral fluids is associated with transmission while detection in blood compartments is associated with disease [13]. However, there are differences in the detection of KSHV and EBV in the different blood compartments. Unlike EBV, KSHV is rarely detected in plasma even among individuals with KS [14]. On the other hand KSHV can be detected in PBMCs and correlates with KS, suggesting that KSHV is majorly cell associated [15, 16]. However, only about 50% of KS patients have detectable KSHV in PBMCs and much less in plasma [17]. Partily because KS is a tumour of endotherial cells (spindal cells) which don’t make part of the PBMCs, and also possibly KSHV infected lymphocytes are mainly in secondary lymphoid organs. KSHV genome copies in PBMC above 10 copies per infected cells represents viral lytic replication, while that below 10 copies per infected cells represents a purely latent infection [17]. Therefore measurement of KSHV in PBMCs is very useful to identify individuals at risk of disease. EBV copies in plasma have been linked to disease and have been shown to better predict EBV disease compared to EBV in PBMCs; however, EBV copies in PBMCs are also increased in EBV associated disease [18, 19]. Therefore EBV in PBMCs could also be very usefull at identifying individuals at risk of disease.

We have previously shown the pattern of KSHV viral load in oral fluids and PBMCs and associated risk factors across a wide age range in HIV-negative individuals from a KSHV endemic area [20]. Furthermore, in a Cameroon KS case–control study, we have shown EBV and KSHV viral load interactions in PBMCs and oral fluids [21]. However, the Cameroon KS study included only adults some of whom were HIV infected and/or had KS. Here we are showing the pattern of EBV and KSHV viral load in PBMCs and oral fluids and associated risk factors across a wide (3–89 years old) age range in HIV-negative individuals from a KSHV endemic area of Uganda [22].

## Methods

### Study design and population

As reported previously [20], this work was carried out within a rural African cohort, the General Population Cohort (GPC). The GPC is a community-based cohort of about 22,000 people in 25 adjacent villages in southwestern Uganda. It was established in 1989 to investigate the epidemiology of HIV; participants from the GPC have been followed ever since. The seroprevalence of KSHV is > 90% in adults [22]. Between July 2017 and November 2017, we nested a cross-sectional study within the GPC enrolling 975 KSHV seropositive (tested previously [13]), HIV-negative individuals aged three to eighty-nine years. Participants were selected randomly after stratification for age and sex. Blood, stool and oral fluids samples were collected from these individuals. Socio-demographic data were collected using standard questionnaires. DNA was extracted from 2 million PBMCs collected and saliva pellets. This DNA was used to quantify both KSHV and EBV. The study was approved by the UVRI-Research and Ethics Committee (REC) (reference number: GC/127/16/09/566), the Uganda National Council for Science and Technology (UNCST) (reference number: HS2123) and LSHTM Ethics Committee (reference number: 11881). Written informed consent was obtained from all adults aged 18 years and above. Parents or guardians consented for children below 18 years, additionally, children aged 8–17 years provided written assent.

Peripheral blood mononuclear cell (PBMC)s and plasma were obtained from the blood for immunological and virological analyses. Study participants were instructed to rinse with 5 mL of Listerine mouthwash, and collect the resulting fluid in a polypropylene tube. Aliquots (of 1 mL each) of oral fluids were spun at 13,000 rcf for 10 min to form oral fluids pellets. Thereafter the supernatant was removed and the oral fluids pellet was stored at − 80 °C. A pellet of two million PBMCs and oral fluids pellets were processed for DNA extraction using a QIAamp blood kit (Qiagen, Valencia, CA), following the manufacturer's instructions.

### EBV real-time PCR

Using DNA extracted previously [13], EBV DNA was quantified in PBMCs and oral fluids from 833 individuals with KSHV viral load data [20]. EBV viral load was quantified using real-time PCR. EBV DNA was amplified using primers (Balf5 EBV forward: 5′-CGG AAG CCC TCT GGA CTT C-3′, - Balf5 EBV reverse: 5′-CCC TGT TTA TCC GAT GGA ATG-3′) and probe (Balf5 EBV Probe: 5′-/56-FAM/TGT ACA CGC ACG AGA AAT GCG CCT/3BHQ_1/-3′) previously reported to be specific to the Balf5 gene [6, 23]. Additionally, B-Actin was amplified in the same sample as an internal positive control using primers (B-Actin forward: 5′-TCA CCC ACA CTG TGC CCA TCT ACG A-3′, B-Actin reverse: 5′-CAG CGG AAC CGC TCA TTG CCA ATG G-3′) and probe (B-Actin Probe: 5′-/5HEX/ATG CCC TCC CCC ATG CCA TCC TGC GT/3BHQ_1/-3′) as previously reported [24].

### KSHV real time PCR

KSHV viral load was quantified using real-time PCR. KSHV DNA was detected using primers (K6 forward primer K6-10F 5′-CGCCTAATAGCTGCTGCTACGG-3′, K6 reverse primer K6-10R 5′-TGCATCAGCTGCCTAACCCAG-3′) and a probe (K6 probe p-K6-10 5′-R-CACCCACCGCCCGTCCAAATTC-Q-3′) previously reported to be specific to the K6 gene region [25]. Additionally, the number of cellular equivalents were determined using a quantitative assay specific to human endogenous retrovirus 3 (ERV-3), which is present in two copies per genomic cell, using these primers (ERV-3 Forward primer PHP10-F 5′-CATGGGAAGCAAGGGAACTAATG′ ERV-3 Reverse primer PHP10-R 5′-CCCAGCGAGCAATACAGAATTT-3′) and a probe (ERV-3 Probe PHP-P505 5′-R-TCTTCCCTCGAACCTGCACCATCAAGTCA-Q-3′). To quantify both ERV-3 and KSHV DNA, seven two-fold serial dilutions of K6 and ERV-3 were made from clone stocks (starting with 1 × 10^6^ dilution to 1 × 10^0^) to form a standard curve on every plate. ERV-3 was cloned into Bluescript II KS vector (Stratagene, La Jolla, CA, USA) KSHV K6 cloned using PCR Topo II vector, Topo TA Cloning kit, Invitrogen, K 4600-40. This procedure has been reported elsewhere [10, 26, 27].

### KSHV and EBV serology

IgG antibody levels were quantified in plasma using ELISA and a multiplex bead-based assay as previously described [13, 28]. K8.1 and LANA/ORF73 recombinant proteins were used to quantify IgG by ELISA. Seropositivity was deifned as reactivity to either K8.1 or ORF73 proteins. Each ELISA plate contained three positive and three negative control sera. The negative control sera was used to set a cut-off value on each plate as previously reported [29]. DNA was extracted from oral fluids and PBMCs of seropositive individuals. Twenty-five KSHV recombinant proteins including ORF73, K10.5, K5, K14, ORF6, ORF11, ORF55, ORF50, ORF60, K3, ORF38, ORF52, ORF59, ORF65, ORF61, ORF18, K11, K8.1, ORF19, ORF25, ORF26, ORF33, ORF37, ORF44 and ORF63 were included in the multiplex bead assay panel with three EBV proteins (EBNA-1, VCA and EA).

### Statistical analysis

Statistical analysis was carried out using STATA version 13 (StataCorp, College Station, Texas USA). Graphs were drawn using STATA and GraphPad Prism version 8. Viral load levels were log_10_ transformed. First, risk factors associated with viral DNA detection (as a categorical outcome variable) in oral fluids and PBMCs, separately, were obtained using logistic regression modelling. Thereafter, risk factors associated with increasing levels of viral DNA (as a continuous outcome variable) in oral fluids and PBMCs, separately, were determined using linear regression modelling. Chi^2^ test, student T-test and one-way ANOVA were used for crude analysis. The false discovery rate (FDR) was used to correct multiple comparisons of antibody data.

## Results

**Fig. 1:**
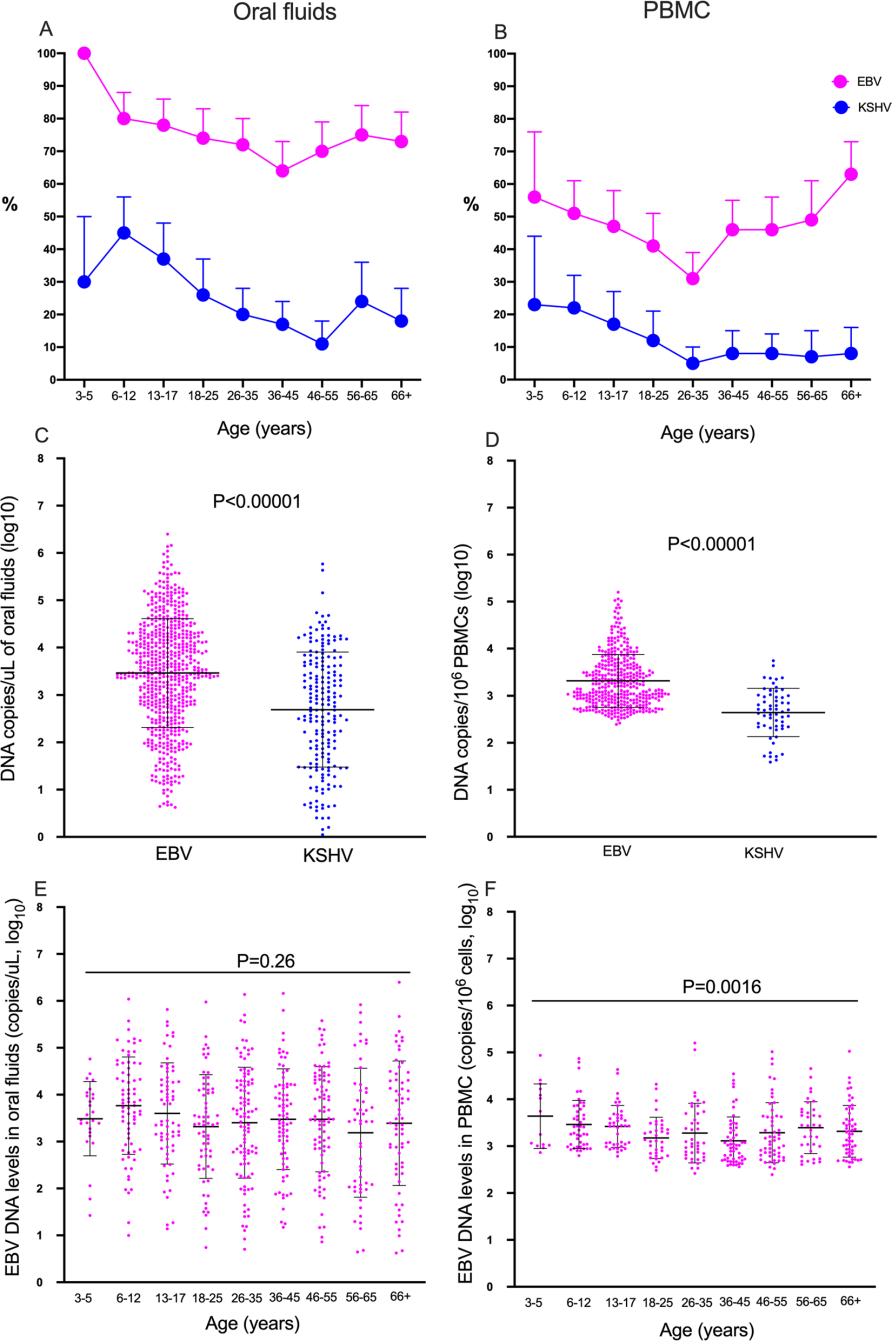
EBV and KSHV in oral fluids and PBMCs. **A** proportion of individuals with EBV or KSHV in saliva; **B** proportion of with EBV or KSHV in PBMCs; **C** EBV and KSHV DNA levels in saliva; **D**: EBV and KSHV DNA levels in PBMCs. EBV viral load in saliva (**E**) and PBMCs (**F**) in each age group. EBV and KSHV Viral load quantified using qPCR. Graphs were drawn in GraphPad Prism version 8. Mean and standard deviation are shown in **C** and **D**. error bars represent 95% Confidence intervals in A and B. Mean and SD are shown in **C**, **D**, E and F. The student T test (**C**, **D**) and Kruskal Wallis test (**E**, **F**) used to obtain P values

Characteristics of the participants included are shown in Table 1. The proportion of individuals with detectable EBV DNA in oral fluids was 74% compared to 24% for KSHV. The median EBV viral load (VL) in oral fluids were 3364 copies/uL while KSHV VL was 401 copies/uL (Table 1). Prevalence of shedding in oral fluids varied with age: all children aged 3–5 years had EBV in oral fluids whereas adults aged 36–45 years had the lowest proportion (72%). For KSHV, the highest proportion with KSHV DNA was among 6–12-year-olds (30%) whereas adults aged 46–55 years old had the lowest (11%) The patterns of KSHV and EBV shedding with age were similar (Fig. 1A).

**Table 1:** Population characteristics and infection status

Age, mean (range)	36 (3–89) years
*Age groups (years)*	
3–5	3% (26/833)
6–12	11% (93/833)
13–17	11% (88/833)
18–25	11% (89/833)
26–35	17% (139/833)
36–45	15% (121/833)
46–55	14% (121/833)
56–65	9% (73/833)
66+	11% (88/833)
Sex, males	49% (409/833)
Malaria parasitaemia^a^	4% (34/833)
EBV DNA levels in saliva (copies/uL)-median (IQR)	3364 (557, 18,860)
KSHV DNA levels in saliva (copies/uL)-median (IQR)	401 (28, 3921)
EBV DNA levels in PBMCs (copies/10^6^ cells)-median (IQR)	1566 (782, 4378)
KSHV DNA levels in PBMCs (copies/10^6^ cells)-median (IQR)	203 (4, 620)
% With detectable EBV in oral fluids	74% (607/824)
% With detectable KSHV in oral fluids	24% (209/874)
% With detectable EBV in PBMCs	46% (377/823)
% With detectable KSHV in PBMCs	11% (94/869)

^a^Asymptomatic malaria by rapid diagnostic test (RDT). Viral load detected using qPCR

Similar to adults from a Cameroon KS case–control study [21], the proportion of individuals with either EBV or KSHV DNA in PBMCs was much lower than the proportion of individuals with either virus in oral fluids (Fig. 1B). Similarly, levels of EBV (median 1566 copies/10^6^ cells) were much higher than levels of KSHV (median 203 copies/10^6^ cells) DNA in PBMCs *P* < 0.00001 (Fig. 1D, Table 1). Overall, 46% of individuals tested had EBV DNA in PBMCs while 11% had KSHV. For both EBV (56%) and KSHV (23%), children aged 3–5 years had the highest (KSHV) and scond highest (EBV) proportions of the virus in PBMCs, while adults aged 26–35 years old had the lowest proportions (EBV: 31%; KSHV: 5% (Fig. 1B). Adults over 66 years of age had the highest proportions of EBV in PBMCs (63% Fig. 1B). levels of EBV DNA in oral fluids were higher than levels of KSHV DNA in oral fluids (Fig. 1C). Children aged 6–12 years had the highest EBV viral load levels in oral fluids, but otherwise, these did not change much across age (Fig. 1E, F).

**Fig. 2:**
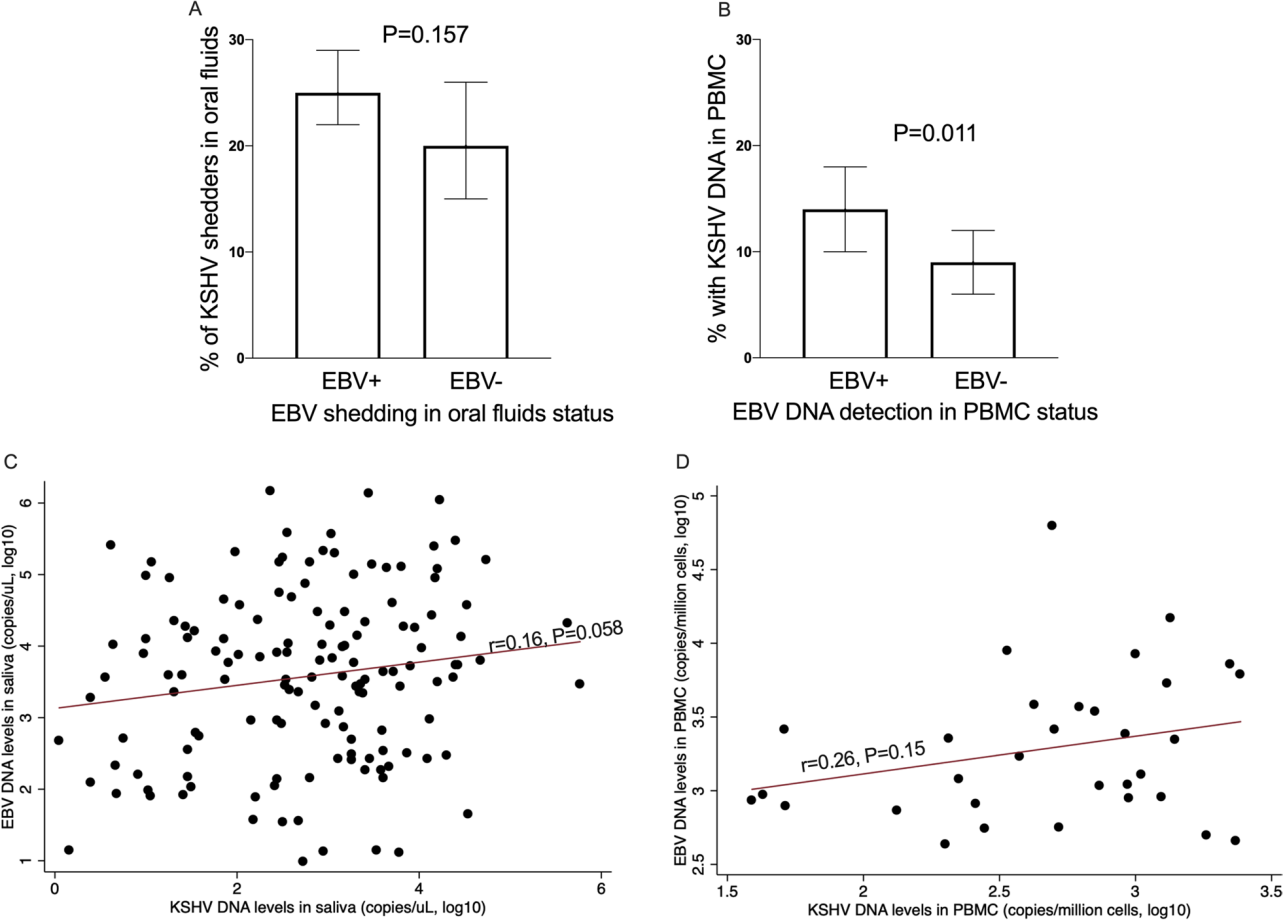
Relationship between KSHV and EBV viral load in oral fluids or PBMCs. **A** proportion of individuals with KSHV in saliva among those with or without EBV in saliva; **B** proportion of with KSHV in PBMCs among those with or without EBV in PBMCs; **C** correlation between EBV and KSHV DNA levels in saliva; **D** correlation between EBV and KSHV DNA levels in PBMCs; EBV and KSHV Viral load quantified using qPCR. Graphs were drawn in GraphPad Prism version 8 (**A**, **B**) and STATA version 13 (**C**, **D**). *P* values and correlation coefficient obtained in STATA version 13. Chi^2^ test used in **A**, and logistics regression adjusting for age and sex in **B**

The proportion of individuals with KSHV DNA in oral fluids did not differ between those with and without EBV DNA in oral fluids (Fig. 2A). However, despite the lower prevalence of either virus in PBMCs compared to oral fluids, the proportion of individuals with KSHV DNA in PBMCS was higher among individuals with EBV DNA in PBMCs (14% vs. 9%, *P* = 0.011) Fig. 2B & Additional file 1: Supplementary Table 1. Both in oral fluids (Fig. 2C) and PBMCs (Fig. 2D), EBV and KSHV DNA levels were positively correlated, although this didn't reach statistical significance.

**Fig. 3:**
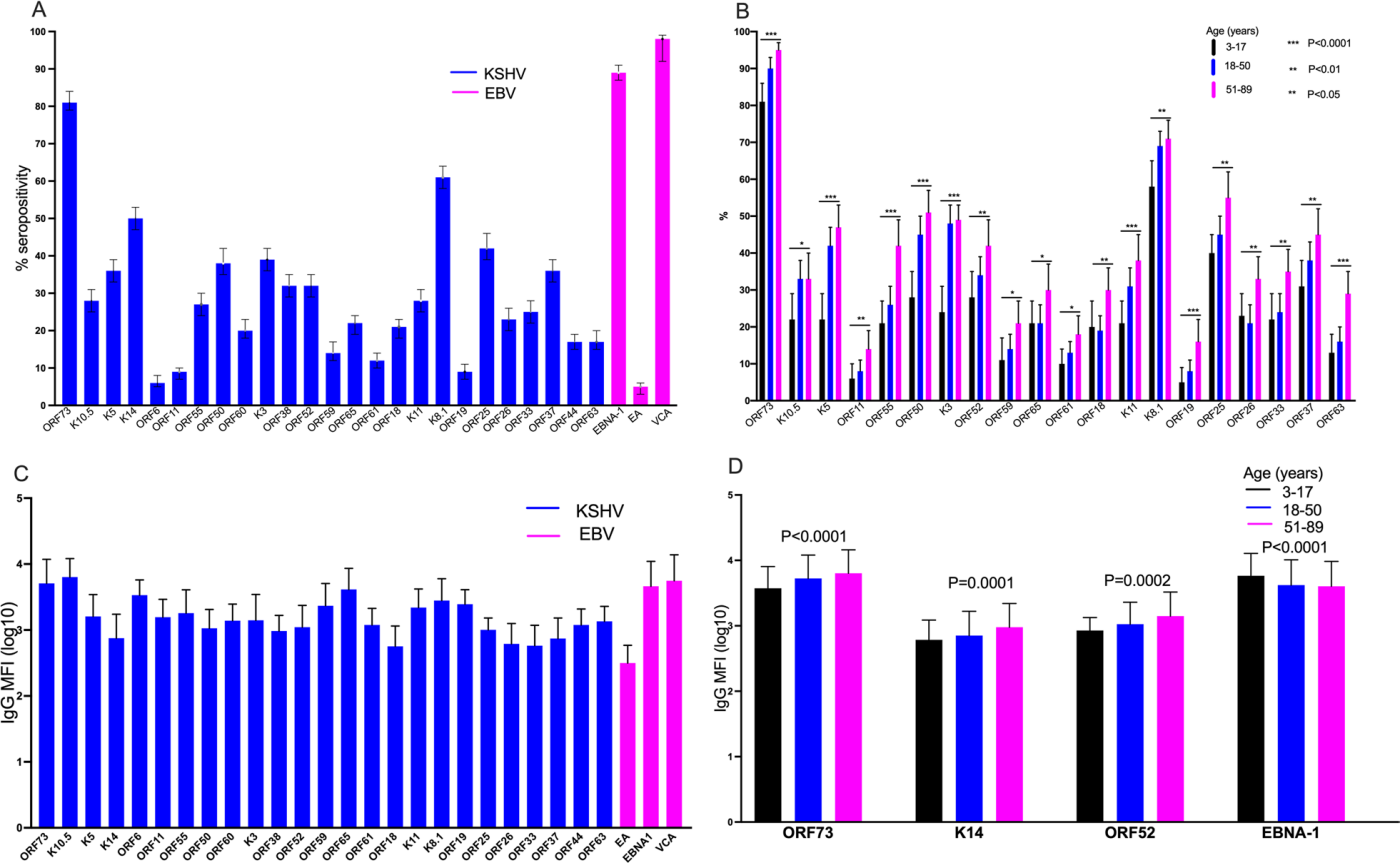
IgG responses to KSHV and EBV proteins. **A** proportion of individuals with a seropositive response to KSHV/EBV proteins. **B** proportion of individuals with a seropositive response to KSHV proteins by age group. **C** mean IgG median fluorescent intensities-MFI to KSHV/EBV proteins. **D** mean IgG MFI to KSHV proteins by age group. Error bars represent standard deviations (**C**, **D**) or 95% confidence intervals (**A**, **B**). *P* value obtained using chi^2^ test (**B**) or one-way ANOVA (**D**) in STATA version 13. Graphs were drawn using GraphPad Prism version 8

**Fig. 4:**
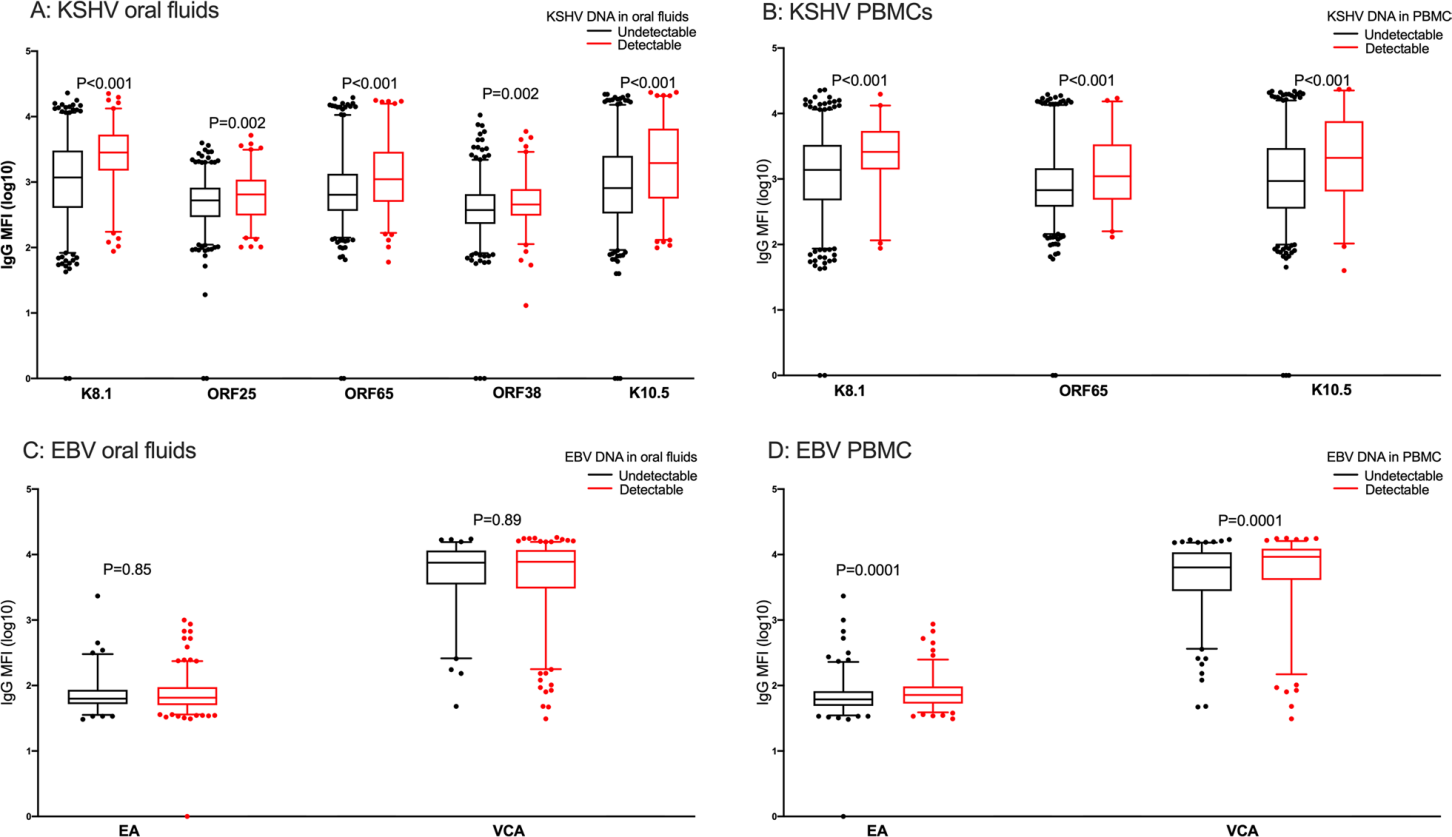
Relationship between viral load and antibody responses to KSHV and EBV. *P* value obtained using a student T-test (KSHV) and Mann–Whitney U test (EBV) in STATA version13. Graphs were drawn in GraphPad Prism version 8. EA: EBV early antigen; VCA EBV viral capsid antigen. ORF25: KSHV major capsid protein; ORF38: KSHV tegument protein; ORF65: KSHV viral capsid protein; K10.5: KSHV viral interferon regulatory factor

Among the 25 KSHV and three EBV proteins used to detect IgG antibody levels in plasma, the majority of the individuals tested responded to ORF73 for KSHV and VCA for EBV (Fig. 3A). The proportion of individuals responding to KSHV ORF73, K10.5, K5, ORF11, ORF55, ORF50, K3, ORF52, ORF59, ORF65, ORF61, ORF18, K11, K8.1, ORF19, ORF25, ORF26, ORF33, ORF37, and ORF63 increased with increasing age (Fig. 3B). Among seropositive individuals, antibody levels to the different KSHV and EBV proteins didn’t differ (Fig. 3C) while antibody levels to the KSHV ORF73, K14 and ORF52 increased with increasing age (Fig. 3D). As we have shown previously [13], IgG antibody levels to K8.1 were higher in individuals with detectable KSHV DNA in PBMCs (Fig. 4A) and in oral fluids (Fig. 4B). Additionally, in comparison to previous findings, IgG antibody levels to ORF65 (a capsid protein) and K10.5 (the viral interferon regulatory factor 3) were also higher in individuals with detectable KSHV DNA in PBMCs (Fig. 4A) and oral fluids (Fig. 4B). Furthermore, IgG antibody levels to ORF25 (a major capsid protein) and ORF38 (a tegument protein) were higher in individuals with detectable KSHV DNA in oral fluids (Fig. 4B).

Age was significantly associated with EBV detection in both oral fluids (*P* = 0.025) and PBMCs (*P* = 0.0089). On the other hand, both *P. falciparum*-infection (detected by malaria rapid diagnostic tests-RDT) and sex were not associated with detection of EBV in oral fluids or PBMCs (Table 2).

**Table 2:** Factors associated with EBV detection in oral fluids or PBMCs

Factor	Oral fluids			PBMCs		
	% With virus	OR (95% CI)*	*P* value	% With virus	OR (95% CI)*	*P* value
*Sex*						
Females	70% (297/422)	1	0.056	44% (183/420)	1	0.226
Males	77% (310/402)	1.36 (0.99, 1.86)		48% (194/403)	1.19 (0.90, 1.57)	
*Age groups (years)*						
3–17	82% (166/203)	1	0.025	50% (101/204)	1	0.0089
18–50	71% (282/397)	0.57 (0.37, 0.87)		40% (159/395)	0.70 (0.50, 0.99)	
51–89	71% (159/224)	0.57 (0.36, 0.91)		52% (117/224)	1.14 (0.78, 1.68)	
*Malaria parasitaemia*^*a*^						
Negative	73% (580/790)	1	0.772	46% (361/789)	1	0.935
Positive	79% (27/34)	1.14 (0.48, 2.70)		47% (16/34)	1.03 (0.51, 2.08)	

*Adjusted for age group, sex and malaria parasitaemia. ^a^Asymptomatic malaria by rapid diagnostic test (RDT). Logistic regression modelling done using STATA version 13. Viral load detected using qPCR

**Fig. 5:**
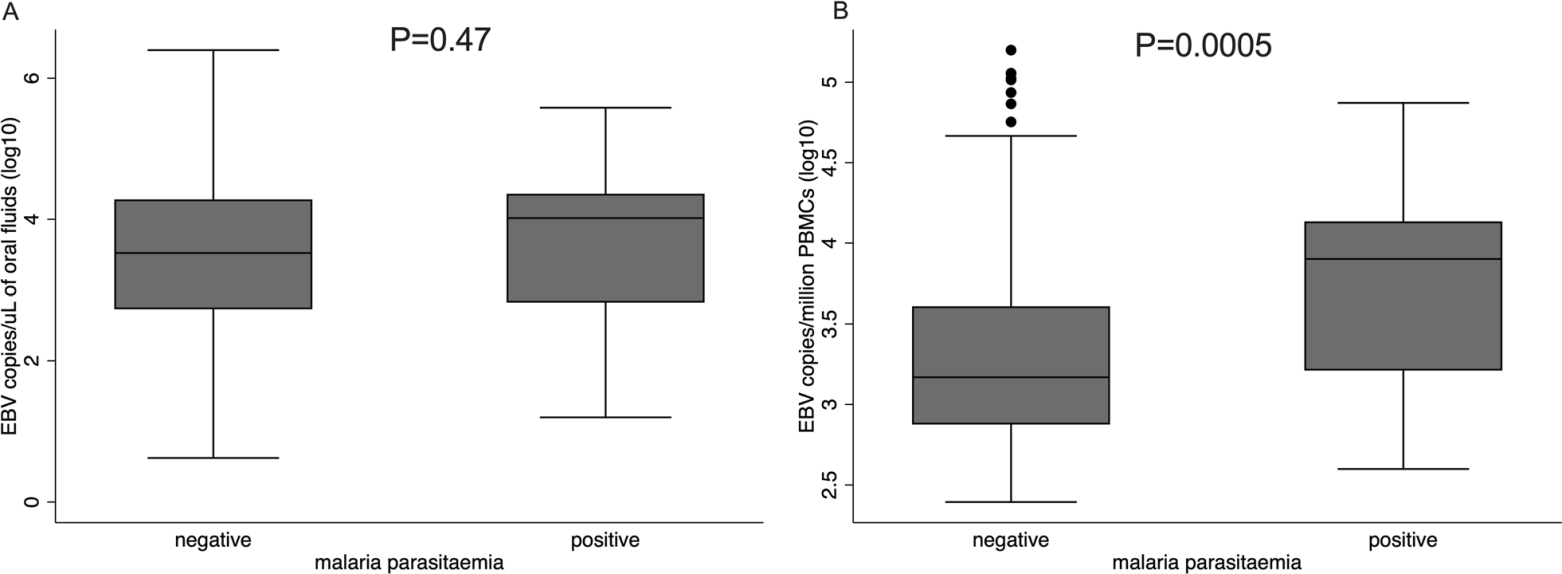
EBV viral copies in oral fluids (**A**) and PBMCs (**B**) by malaria parasitaemia status. *P* values obtained from a student T test. Malaria parasitaemia determined using Rapid diagnostic tests (RDT). EBV quantified in oral fluids and PBMCs using qPCR

Among those with EBV in oral fluids, malaria and sex were not associated with EBV levels. However, among those with EBV in PBMCs, malaria was positively associated with EBV DNA levels. Individuals with malaria had higher levels of EBV DNA in PBMCs compared to individuals without malaria (adjusted regression coefficient 0.43, (0.15–0.71), *P* = 0.002). Sex was not associated with levels of EBV DNA in PBMCs. Age group was associated with levels of EBV DNA in both oral fluids and PBMCs (Table 3, Fig. 5).

**Table 3:** Factors associated with EBV viral load among individuals with detectable EBV in oral fluids or PBMCs

Factor	Oral fluids		PBMCs	
	Coefficient (95% CI)*	*P* value	Coefficient (95% CI)*	*P* value
*Sex*				
Females	Ref		Ref	
Males	0.06 (− 0.13, 0.24)	0.550	− 0.05 (− 0.16, 0.06)	0.348
*Age groups (years)*				
3–17	Ref		Ref	
18–50	− 0.23 (− 0.45, − 0.004)		− 0.26 (− 0.40, − 0.12)	
51–89	− 0.31 (− 0.56, − 0.05)	0.046	− 0.08 (− 0.22, 0.07)	0.0004
Malaria parasitaemia^a^				
Negative	Ref		Ref	
Positive	0.05 (− 0.40, 0.50)	0.826	0.43 (0.15, 0.71)	0.002

*Adjusted for age group, sex and malaria parasitaemia. ^a^Asymptomatic malaria by rapid diagnostic test (RDT). Logistic regression modelling done using STATA version 13. Viral load detected using qPCR

## Discussion

This study presents the following observations: (1) both in oral fluids and PBMCs, EBV is detected more frequently and in higher quantities compared to KSHV, as shown previously [21]; (2) both viruses are more likely to be detected in children's oral fluids and in PBMCs than in other age groups; (3) Individuals with KSHV in PBMCs are more likely to have EBV in PBMCs as well; (4) Infection with asymptomatic *P. falciparum* malaria is associated with higher EBV viral load in PBMCs; (5) in addition to IgG antibody levels to the KSHV K8.1, IgG antibody levels to KSHV ORF65 and K10.5 are higher in individuals with detectable KSHV in PBMCs and oral fluids while IgG antibodies to the KSHV ORF38 and ORF25 are higher in individuals with detectable KSHV in oral fluids only.

EBV is ubiquitous in all human populations with over 90% of adults infected [30] whereas KSHV is limited to specific areas or high-risk populations, most notably in sub-Saharan Africa [31]. The findings suggest that EBV is more easily transmitted than KSHV. The mechanism leading to the difference in transmissibility between the two viruses is not documented. This study and previous studies showing that EBV DNA is more frequently detected in oral fluids and at higher levels than KSHV contributes to our understanding of the differences in transmission patterns between the two viruses. The difference cannot be solely explained by cell tropism because both KSHV and EBV infect several types of cells, some of which overlap. EBV infects B lymphocytes, epithelial cells, T lymphocytes, NK cells, monocytes, smooth muscle cells and follicular dendritic cells using CD21, HLA-II, integrins and EphA2 for attachment, internalization and entry [32]. KSHV infects endothelial cells, fibroblasts, monocytes, epithelial cells, B lymphocytes, macrophages and dendritic cells using HSPGs, DC-SIGN, EphAs and integrins for attachment and entry [33].

EBV and KSHV are more likely to be detected in children compared to adults. This might be attributed to a more recent infection with the viruses. Viral control may have not been well established in children and could be developed over time as individuals age. Furthermore, the high burden of malaria infection in children could be driving viral reactivation of KSHV and EBV. We observed that individuals with detectable KSHV in PBMCs are more likely to have detectable EBV as well, we speculate that systemic factors affecting viral immune control including immunosuppression, Th2 skew, immune regulation, and immune cell dysfunction could affect the control of both viruses.

Epidemiology studies have linked EBV and *P. falciparum* to Burkitt’s lymphoma [34]. Both EBV and *P. falciparum* upregulate AID expression while AID expression has been shown to contribute to c-MYC translocation and mutation [35–37]. c-MYC translocation is a hallmark of Burkitt lymphoma development [36]. Additionally, chronic exposure to *P. falciparum* has been shown to reactivate EBV, increasing the number of latently infected B cells with EBV [38]. Possibly *P. falciparum* impairs T cell immunity to EBV through immune suppression leading to EBV viral reactivation hence increasing the number of B cells infected by EBV. We have previously shown a similar association between *P. falciparum* infection and increased KSHV viral load [20]. The current finding that detection of EBV increases the risk of detecting KSHV in PBMCs, and the similar association of EBV and KSHV viral load in PBMCs with malaria infection, suggests that malaria could be affecting EBV and KSHV by causing immune dysfunction leading to viral reactivation of both viruses.

The strength of this study was the large sample size (over 800 individuals analysed) and the inclusion of males and females across the life course (3–89 years). Furthermore, all individuals analysed were HIV uninfected, so the impact of HIV on viral reactivation is not a concern. Although HIV has been shown to dramatically reactivate both viruses, in endemic regions, transmission of both viruses occurs in childhood before HIV acquisition for most individuals. The major weakness of this study is the cross-sectional design of the study.

## Conclusion

EBV is more frequently detected and at higher levels, both in PBMCs and oral fluids than is KSHV. Viral detection of both KSHV and EBV is more frequent in children compared to adults. This might in part be explained by the burden of *P. falciparum* infection in children and the recent viral infection. The mechanism through which *P. falciparum* affects both KSHV and EBV warrants further investigation.

## Supplementary Information

**Additional file 1.** Supplementary Table 1 shows crude and adjusted associatiations between detection of KSHV anddetection of EBV DNA in PBMCs.

## Data Availability

Data supporting the findings of this study are available within the manuscript and its supplementary material. Raw data that supports the findings of this study are available from the corresponding author upon request.
