# Supplementary material for: Comparison of Epstein–Barr virus and Kaposi’s sarcoma-associated herpesvirus viral load in peripheral blood mononuclear cells and oral fluids of HIV-negative individuals aged 3–89 years from Uganda
Source: Infect Agent Cancer. 2023 Jun 14;18:38. doi: 10.1186/s13027-023-00516-9 (PMC10268376; doi:10.1186/s13027-023-00516-9)
Supplement: Supplementary file 1 — Additional file 1. Supplementary Table 1 shows crude and adjusted associatiations between detection of KSHV anddetection of EBV DNA in PBMCs. [file 13027_2023_516_MOESM1_ESM.docx]

Supplementary Table 1: Association between detection of KSHV and EBV DNA in PBMCs.

|  |  | Un adjusted | | Adjusted for age and sex | |
| --- | --- | --- | --- | --- | --- |
|  | Detectable KSHV in PBMCs | OR (95% CI) | P value | OR (95% CI) | P value |
| undetectable EBV in PBMCs  detectable EBV in PBMCs | 9% (38/445)  14% (52/377) | 1  1.7 (1.1-2.7) | 0.017 | 1  1.8 (1.1 – 2.8) | 0.011 |

EBV and KSHV Viral load quantified using qPCR. Logistic regression used for statistical analysis in STATA version 13
